# Optimization of process parameters for preparation of polystyrene PM2.5 particles by supercritical antisolvent method using BBD-RSM

**DOI:** 10.1038/s41598-020-67994-4

**Published:** 2020-07-07

**Authors:** Zhuo Zhang, Qingling Li, Bo Guo, Shouzhong Zhang, Sen Zhang, Dedong Hu

**Affiliations:** 1 School of Electromechanical Engineering, Qingdao University of Science and Technology, Qingdao, 266061 China; 2 Shandong Institute of Metrology, Jinan, 250014 China; 3 Shandong Zhifu District Market Supervision Administration, Yantai, 264001 China

**Keywords:** Design, synthesis and processing, Experimental particle physics

## Abstract

The objective of this study is to optimize the process parameters for preparing polystyrene (PS) PM2.5 particles by supercritical antisolvent (SAS) method. Toluene was selected as the solvent and supercritical carbon dioxide (SC-CO_2_) was used as the antisolvent. The Box–Behnken design-response surface method was applied to investigate the effect of crystallizer pressure, PS massic concentration, flow ratio of CO_2_/solution and crystallizer temperature on the size and the distribution of PS particles, systematically. It is found that crystallizer temperature is the most significant variable on the size and the distribution of PS particles, followed by flow ratio of CO_2_/solution and PS massic concentration, and crystallizer pressure is the slightest significant factor. The particle size increases with the increase of crystallizer temperature. The optimum conditions are obtained as crystallizer pressure 9.8 MPa, PS massic concentration 1.6 wt%, flow ratio of CO_2_/solution 140 g/g and crystallizer temperature 309 K. Under these conditions, the PS particle with the size of 2.78 μm and a narrow size distribution has been prepared, meeting PM2.5 standard aerosols. The results suggest that it is feasible to produce PM2.5 standard aerosols by SAS.

## Introduction

Polystyrene (PS), the standard of calibration of optical particle counter, is of vital importance for the PM2.5 monitor. Due to colorless, tasteless, dissolved in organic solvents and stabilized in physicochemical properties, it is considered as the first choice for the preparation of PM2.5 aerosol^1–5^. Recently, there have been great interests in finding environment-friendly and reliable means of producing fine PS particles for calibrating in PM2.5 monitor^6^.

Traditional PS PM2.5 aerosol preparing processes such as fluidized bed^7^, atomization^8^ and agglutination^9^ cannot meet the requirement of verifying and calibrating the PM2.5 monitor because of the difficulty in aerosol particle size control and wide particle size distribution. Supercritical fluid technology had been widely applied to produce micro and nanoparticles in various fields such as chemical, materials and pharmaceutical^10–14^. It exhibits its superiority including high product quality, environment-friendly properties, low cost, solvent free and easy control on product. Supercritical antisolvent (SAS) process has been selected as the priority method since most organic compounds are insoluble or slightly soluble in supercritical fluid. Many studies have shown that the most prominent characteristic of SAS process is that it can lead to particles with small size, unique morphology, narrow size distribution and lower residual solvent. Additionally, supercritical carbon dioxide (SC-CO_2_) has been the most popular solvent as its advantages like non-toxic, non-flammable, economical, evaporates residue-free at ambient pressure^15,16^.The particle precipitation mechanisms between mass transfer, hydrodynamic and phase equilibrium of the system, as well as kinetics of nucleation and growth were discussed in many papers^17–20^.

It is well-known that numerous investigations about PS particles prepared by SAS process have been conducted previously. Jeong et al.^21^ obtained PS particles ranged from 10^2^ to 10^4^ nm using dichloromethane as solvent by aerosol solvent extraction system. By employing chloroform as solvent, Reverchon et al.^22^ obtained PS aerosol by SAS process to form solid bridge nanoparticles. Santiago et al.^23^ prepared polymeric matrix of PS by SAS process using ethyl acetate as solvent and SC-CO_2_ as antisolvent and obtained two groups of nearly spherical sub-microns particles with a particle size between 150 and 400 nm. The results demonstrated that it is feasible to acquire PS particles by SAS process, but the PS PM2.5 standard particles have not been prepared.

The Box–Behnken design-response surface method (BBD-RSM) is a second-order experimental design method based on three levels. It can evaluate the non-linear relationship between indexes and factors to optimize the operating conditions for responses affected by multiple variables^24,25^. The objective of this research is to optimize the conditions for preparing PS PM2.5 particles by SAS process based on BBD-RSM. Toluene was selected as a solvent and SC-CO_2_ was used as an antisolvent. Further more, the effects of crystallizer pressure, PS massic concentration, flow ratio of CO_2_/solution and crystallizer temperature on the morphology, the size and distribution of the PS particles were investigated systematically by BBD-RSM.

## Materials and methods

### Materials

Carbon dioxide, with purity of 98%, was purchased from Jinan German Foreign Specialty Gases Co., Ltd, China. Polystyrene, with purity of 99.9%, was provided by Tianjin Damao Chemical Reagent Co., Ltd, China. Toluene, with purity of 98%, was purchased from Jinan Xinwang Chemical Co., Ltd, China.

### Apparatus

**Figure 1:**
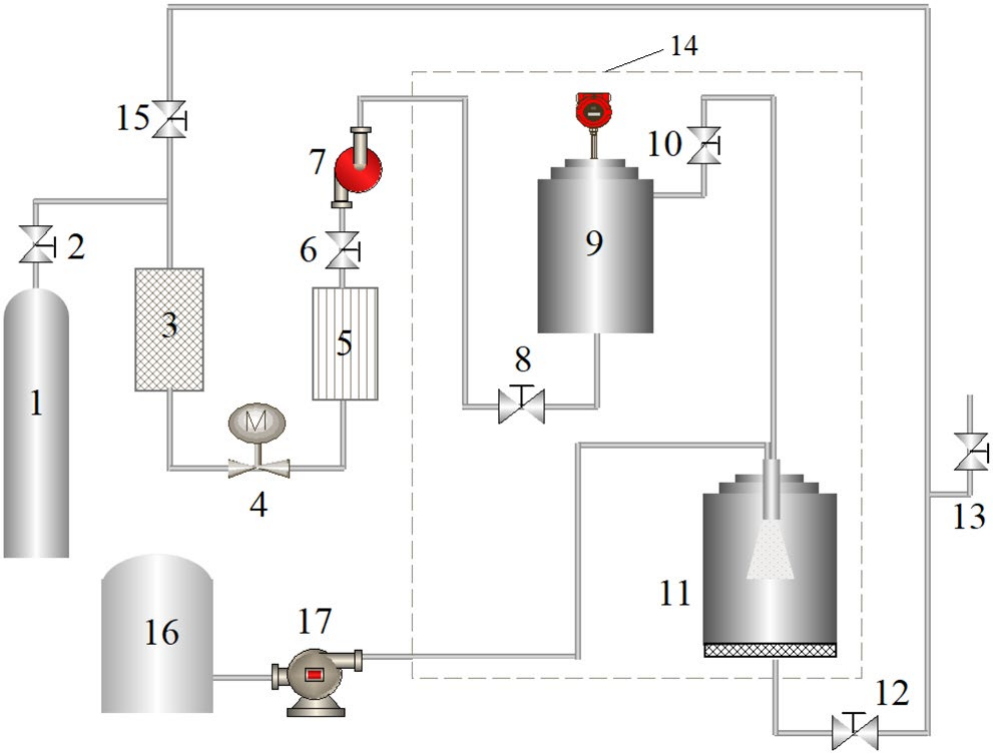
The schematic diagram of the SAS equipment. (1) Carbon dioxide cylinder; (2, 6, 8, 10, 12, 13, 15) screw valve; (3) purifier; (4) flow-meter; (5) refrigeration equipment; (7) plunger pump; (9) buffer vessel; (11) crystallizer; (14) thermostated air chamber; (16) solution tank; (17) advection pump.

**Figure 2:**
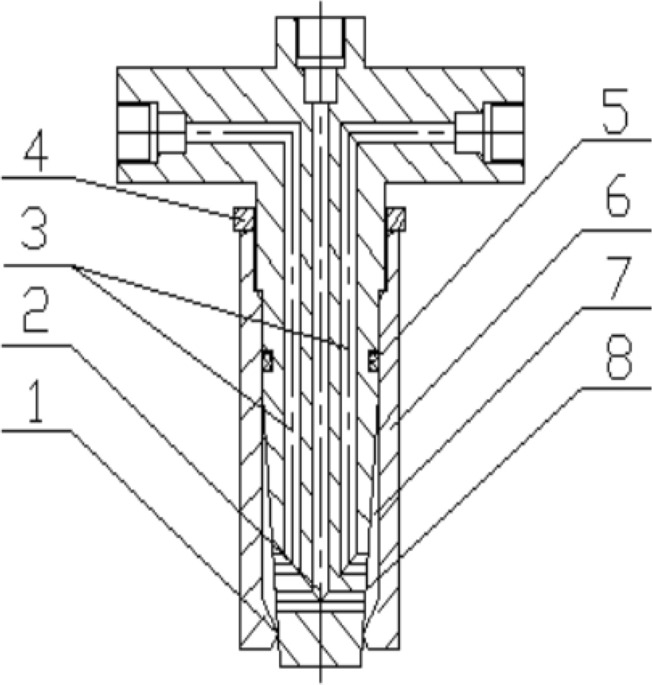
Diagram of coaxial three-channel annular size-adjusting nozzle. (1) Outlet; (2) central pipeline; (3) bypass pipeline; (4) lock nut; (5) seal ring; (6) taper sleeve; (7) mixing chamber; (8) core.

The SAS experiments were carried out in the SAS equipment developed by our team independently^26^. Figure 1 shows the schematic diagram of the apparatus and the structure of the nozzle is presented in Fig. 2. The apparatus was composed of four parts: carbon dioxide supplying unit (consists of carbon dioxide cylinder 1, purifier 3, refrigeration equipment 5, plunger pump 7 and buffer vessel 9 with 500 ml volume), solution delivering unit (consists of solution tank 16 and advection pump 17), particle preparing unit (consists of the coaxial three-channel annular size-adjusting nozzle with diameter 100 μm, heater and crystallizer 11 with 500 ml volume) and auxiliary unit (consists of the flow-meter 4, screw valve, pressure gauge with precision ± 0.5 MPa, thermometer with precision ± 0.1 K, the thermostat air chamber 14 and et al.).

### Experimental procedure

The SAS process was initiated by heating CO_2_ to the desired temperature using a thermostat air chamber 14. In this process, CO_2_ was flowed through a refrigeration equipment 5 to ensure that it was in the liquid phase to prevent pump cavitation. Then, CO_2_ was delivered to the crystallizer 11 via a stainless steel buffer vessel 9 by a plunger pump 7. Screw valve 13 was used to maintain the system pressure by controlling the valve openings. After the desired operating conditions were reached, the PS solution was sprayed into the crystallizer 11 at a flow rate of 10 ml/min through the coaxial three-channel annular size-adjusting nozzle. Once the solution contacted with SC-CO_2_ inside the crystallizer 11, the solute was supersaturated and particles formed. After the aimed amount of solution was processed, SC-CO_2_ was continuously pumped into the crystallizer 11 for at least 60 min to remove the residual solvent completely. If the final purge with SC-CO_2_ is not performed, the solvent may condense during the depressurization step and can solubilize or modify the precipitates. Finally, CO_2_ was slowly vented from the precipitator until the pressure down to atmospheric pressure and the particulate samples were then collected on a metal filter at the bottom of the crystallizer. The various experimental conditions studied here are summarized in Table 1.

**Table 1:** Experimental range and levels of the independent test variables.

Variables	Factor	Range and level		
		− 1	0	1
Crystallizer pressure (MPa)	A	8.5	9.5	10.5
PS massic concentration (wt%)	B	1.0	1.5	2.0
Flow ratio of CO_2_/solution (g/g)	C	92	138	185
Crystallizer temperature (K)	D	308	323	338

### Design of experiments

In order to get better understanding of the process, a design of experiment calculation was utilized to determine the optimal operating conditions. The BBD-RSM is a statistical method in analytical optimization that describes the behavior data set based on fitting a multiple quadratic regression equation to the experimental data. In this model, the independent variables like crystallizer pressure (A), PS massic concentration (B), flow ratio of CO_2_/solution (C) and crystallizer temperature (D) were selected as critical process parameters based on preliminary experiments and references. Each variable varied over three levels, according to the experimental plan shown in Table 1, resulting in 29 experimental runs in total. The behavior of the process was explained by the quadratic polynomial equation as follow:

1 $$Y = \beta_{0} + \sum \beta_{i} x_{i} + \sum \beta_{ij} x_{i} x_{j} + \sum \beta_{ii} x_{i}^{2}$$

where *Y* denotes the value of the particle size, whereas *β*_0_, *β*_*i*_, *β*_*ii*_ and *β*_*ij*_ represent the regression coefficient for the term intercept, linear, square and interaction effects, respectively. Also, *x*_*i*_ and *x*_*j*_ are the independent variables^27,28^.

### Characterization of precipitates

#### Scanning electron microscopy

The morphology of processed particles was examined by NOVA NANOSEM 4323 (Netherlands FEI Co., Ltd.) Scanning Electron Microscopy (SEM). PS particles were spread on the test bench with double-coated adhesive tape and coated with gold by a sputter coater, then put in the SEM and observed at different magnifications.

#### Laser particle size analyzer

Laser Particle Size Analyzer (Winner2000ZD, Jinan Weiner Particle Instrument Co., Ltd.) was employed to analyze the overall distribution of particles in each size segment and make a corresponding distribution histogram.

## Results and discussion

### Optimization of SAS process by BBD

A four-factor with three-level by BBD-RSM was executed to understand the response of the SAS process. Total 29 experimental results of precipitated PS particles obtained are shown in Table 2, indicating that the particle size ranged from 1.2 to 11.0 μm. The second-order polynomial [Eq. (2)] obtained by multi-regression analysis of experimental data represents the mathematical relationship between examined variables and response (PS particle size).

2 $$\begin{aligned} Y & = {7}.{87} - 0.{323}A + 0.{86}B - {1}.{48}C + {3}.{61}D + 0.{34}AB + 0.{63}AC - 0.{89}AD + 0.{15}AC + 0.{55}BD \\ & \quad - {1}.{25}CD + 0.0{58}A^{{2}} - 0.{37}B^{{2}} - {1}.{18}C^{{2}} - {2}.0{5}D^{{2}} \\ \end{aligned}$$

**Table 2:** Design and experimental results of response surface analysis.

Run	Crystallizer pressure (MPa)	PS massic concentration (wt%)	Flow ratio of CO_2_/solution (g/g)	Crystallization temperature (K)	Average particle size (μm)	
					Experimental	Predicted
1	8.5	1.5	92	323	8.01	9.36
2	9.5	1.5	138	323	8.31	7.87
3	10.5	1.5	92	323	6.03	7.10
4	9.5	1.5	138	323	6.05	7.87
5	9.5	1.5	92	308	2.11	1.26
6	9.5	1.5	92	338	11.01	10.99
7	9.5	1.5	185	308	2.01	0.80
8	10.5	1.5	185	323	6.46	5.39
9	9.5	1.0	185	323	2.38	3.83
10	8.5	2.0	138	323	9.52	8.58
11	10.5	1.5	138	338	7.96	8.11
12	9.5	1.0	138	338	7.51	7.66
13	8.5	1.5	138	308	1.08	1.89
14	9.5	1.0	138	308	1.21	1.53
15	8.5	1.5	138	338	10.73	10.89
16	9.5	1.5	185	338	5.92	5.53
17	9.5	2.0	138	338	10.52	10.48
18	9.5	1.0	92	323	8.14	7.09
19	10.5	1.5	138	308	1.86	2.66
20	8.5	1.0	138	323	8.12	7.54
21	9.5	2.0	138	308	2.03	2.16
22	9.5	1.5	138	323	8.03	7.87
23	10.5	1.0	138	323	6.17	5.87
24	9.5	2.0	185	323	3.84	5.85
25	8.5	1.5	185	323	5.93	5.14
26	9.5	2.0	92	323	9.01	8.51
27	10.5	2.0	138	323	8.91	8.25
28	9.5	1.5	138	323	8.02	7.87
29	9.5	1.5	138	323	8.96	7.87

**Figure 3:**
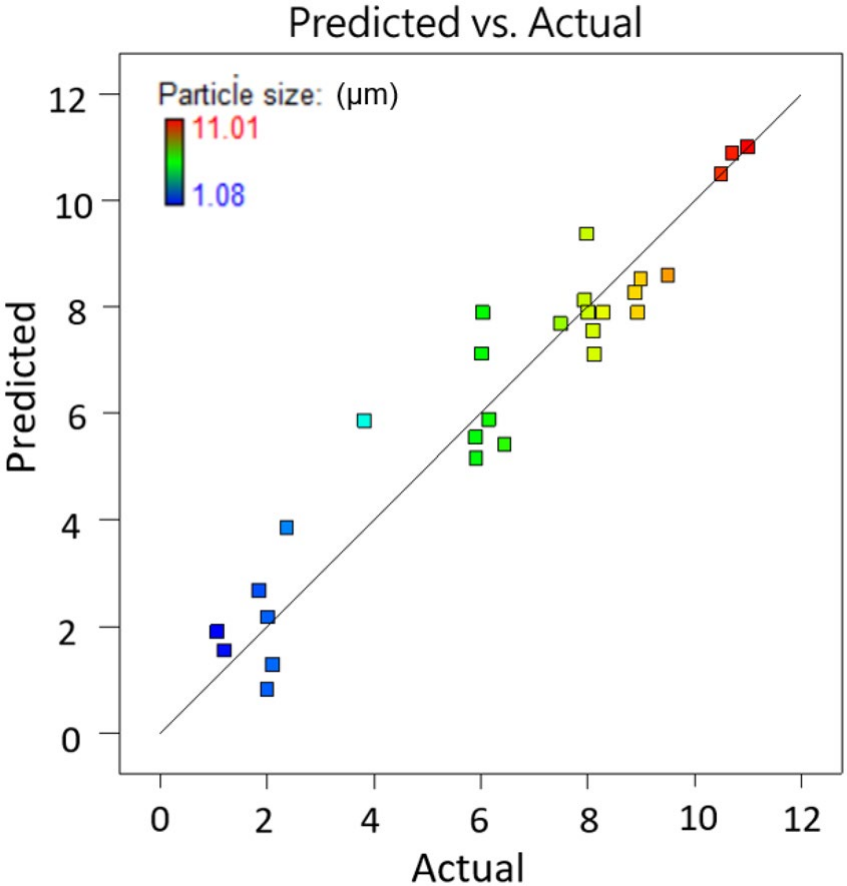
The relationship of predicted and actual values of the model.

**Figure 4:**
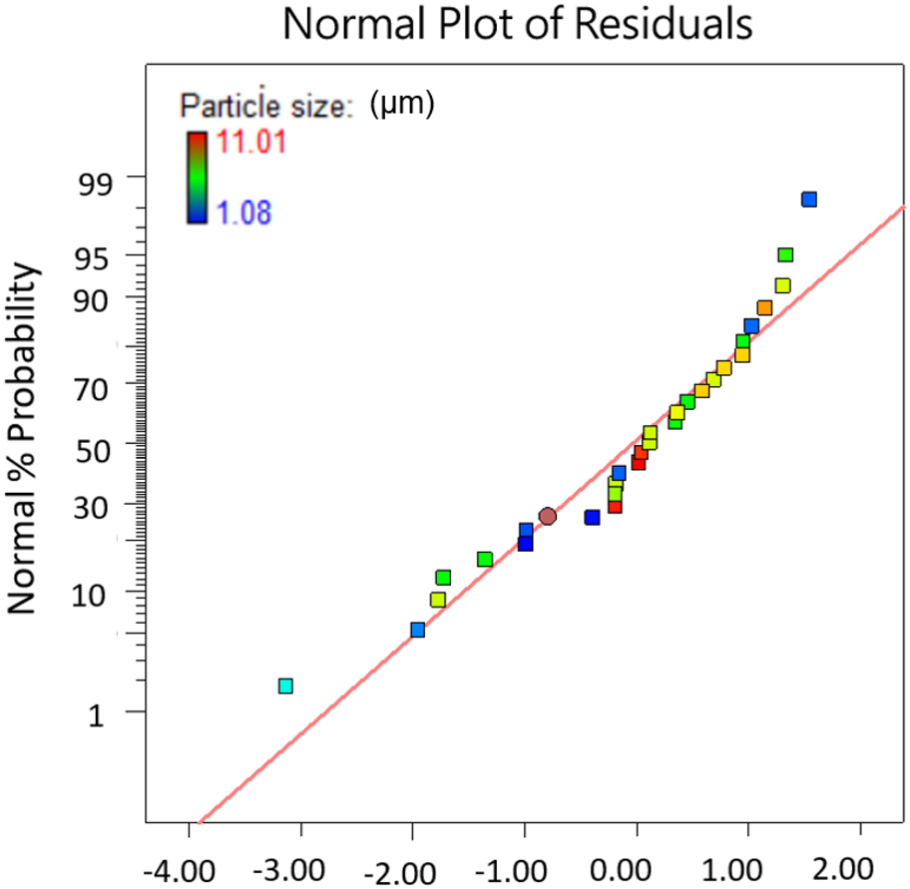
The normal % probability distribution versus externally studentized residuals.

**Figure 5:**
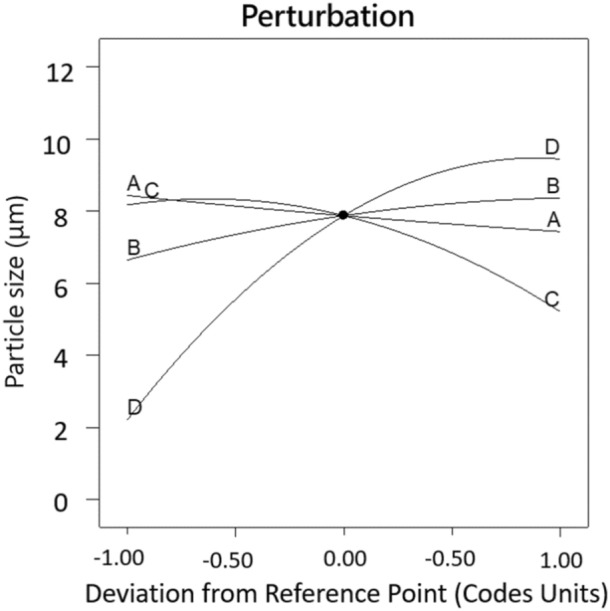
Perturbation plots for the PS particle size. (**A**) Crystallizer pressure; (**B**) PS massic concentration; (**C**) flow ratio of CO_2_/solution and (**D**) crystallizer temperature.

The independence and interaction of variables on the response and significant results are assessed using analysis of variance (ANOVA) (Table 3). The *P* value is used to check the significance of each coefficient and interaction strength between each independent variable. The *F* and *P *value of the model are 10.72 and < 0.0001, respectively, indicating that regression model of PS particle size is highly significant. The coefficient of determination *R*^2^ of 0.930 also reveals a good correlation between the actual and the predicted values as shown in Fig. 3. The adequate precision of this model is 12.377, which is greater than 4, indicating that the model is desirable. Obviously, the points on the residual normal graph (Fig. 4) are close to the straight line, which indicates the accuracy of the model, as well as the independence of the residuals. As shown in Table 3 that the linear model terms (*B*, *C* and *D*), quadratic model terms (*C*^2^ and *D*^2^) are statistically significant for PS particle size, while other interactions are proven to be not significant. Obviously, crystallizer temperature is the most significant variable for the response with the smallest *P* value of < 0.0001 but crystallizer pressure does not have an influence on the particle size with the highest *P* value among the four variables. The same conclusion can be drawn from Fig. 5 that four parameters have significant effects in the order (crystallizer temperature > flow ratio of CO_2_/solution > PS massic concentration > crystallizer pressure) on particle size.

**Table 3:** Analysis of variance (ANOVA) results of the regression equation.

Source	Sum of Squares	Degree of freedom	Mean square	*F*-value	*P* value Prob > *F*
Model	241.15	14	17.23	10.72	< 0.0001
A	3.00	1	3.00	1.87	0.1934
B	8.84	1	8.84	5.5	0.0343
C	26.31	1	26.31	16.37	0.0012
D	156.60	1	156.60	97.45	< 0.0001
AB	0.45	1	0.45	0.28	0.6054
AC	1.58	1	1.58	0.98	0.3390
AD	3.15	1	3.15	1.96	0.1832
BC	0.087	1	0.087	0.054	0.8194
BD	1.20	1	1.20	0.75	0.4023
CD	6.23	1	6.23	3.87	0.0692
A^2^	0.022	1	0.022	0.013	0.9095
B^2^	0.89	1	0.89	0.55	0.4697
C^2^	9.09	1	9.09	5.66	0.0322
D^2^	27.16	1	27.16	16.90	0.0011
Residual	22.323	14	1.61		
Pure error	4.74	4	0.17		
Cor total	263.338	28			
SD	1.15		R^2^		0.930
Mean	6.47		Adj-R^2^		0.8608
C. V. (%)	17.75		Pred-R^2^		0.6100
Press	103.36		Adequate precision		12.337

*Significance at *P* < 0.05.

The optimal conditions (9.72 MPa, 1.59%, 141.89 g/g and 309.18 K) was obtained by Design Expert Software 8.06 theoretically. The particle size predicted by this model was reduced to 2.5 μm. Validation of the design was performed by confirmatory experimental in order to examine the consistency between the theory and reality. Considering the practicality of operation, the optimal conditions were set at crystallizer pressure 9.8 MPa, PS massic concentration 1.6 wt%, flow ratio of CO_2_/solution 140 g/g and crystallizer temperature 309 K. After three repeated experiments, average particle size of PS particles was 2.78 μm, similar to the theoretical predicted value.

### Effect of operating conditions of SAS on the particles size

**Figure 6:**
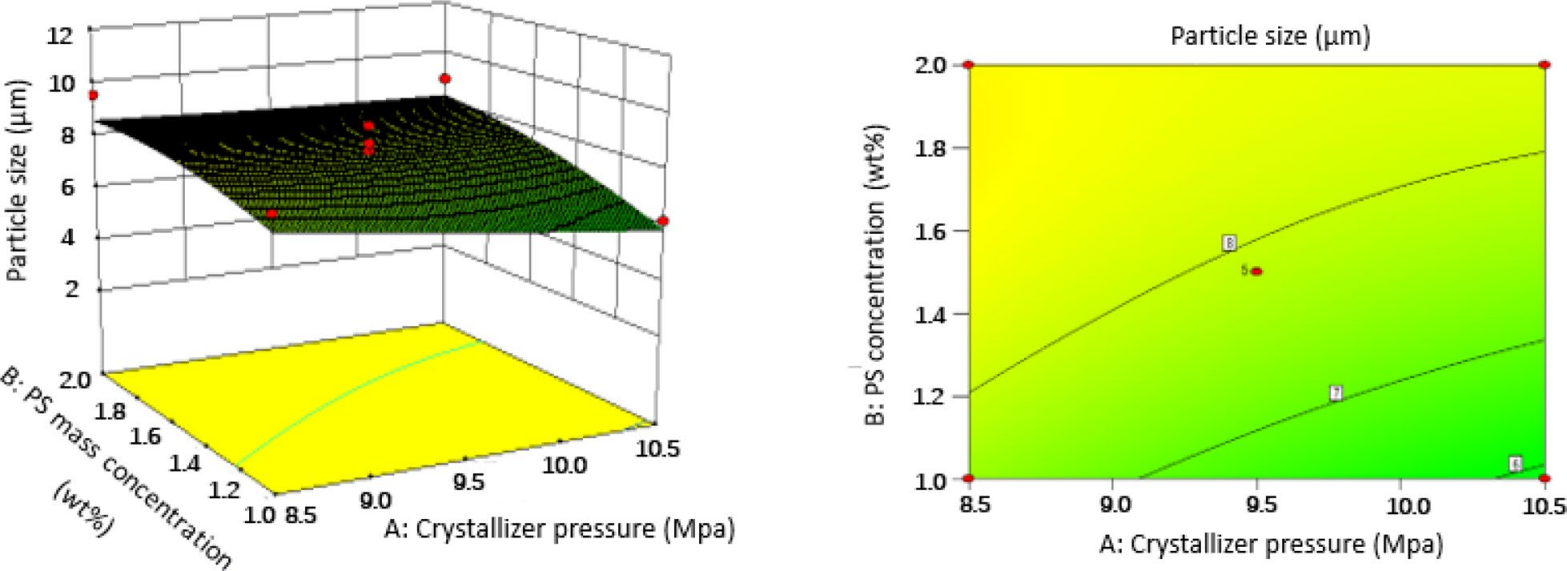
3D response surface and 2D contour plots showing the interaction between (**A**) crystallizer pressure and (**B**) PS massic concentration.

**Figure 7:**
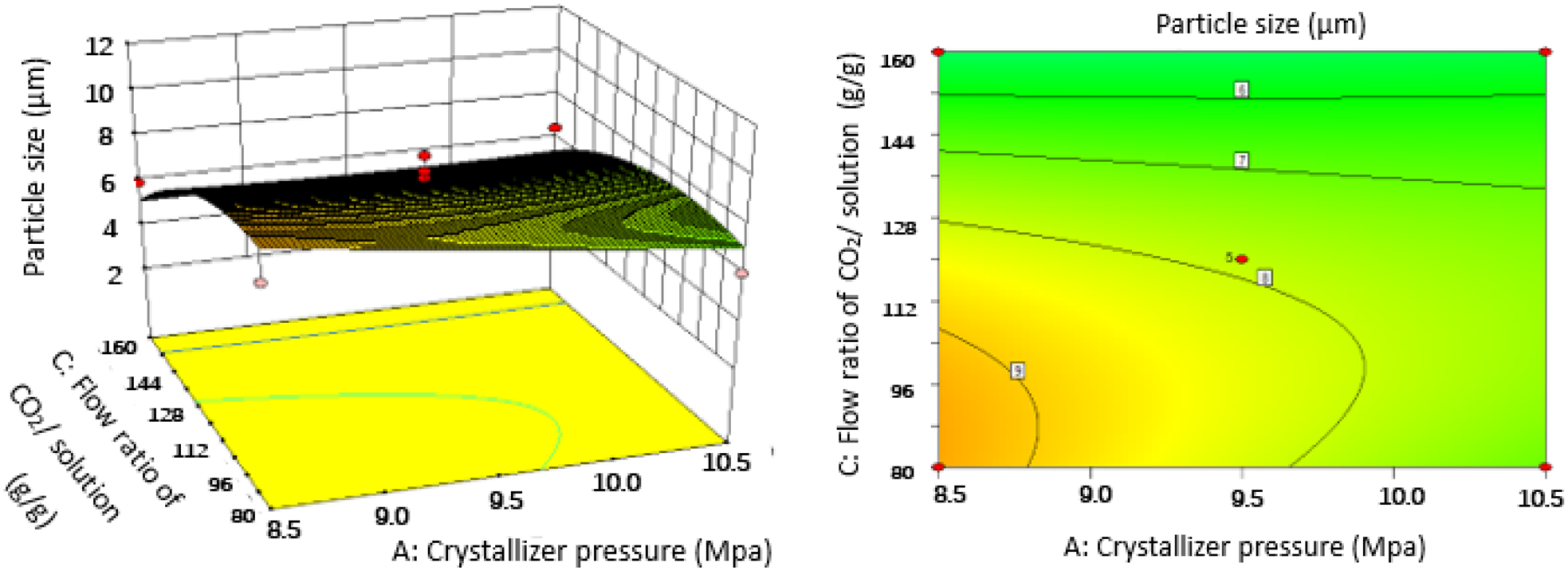
3D response surface and 2D contour plots showing the interaction between (**A**) crystallizer pressure and (**C**) flow ratio of CO_2_/solution.

**Figure 8:**
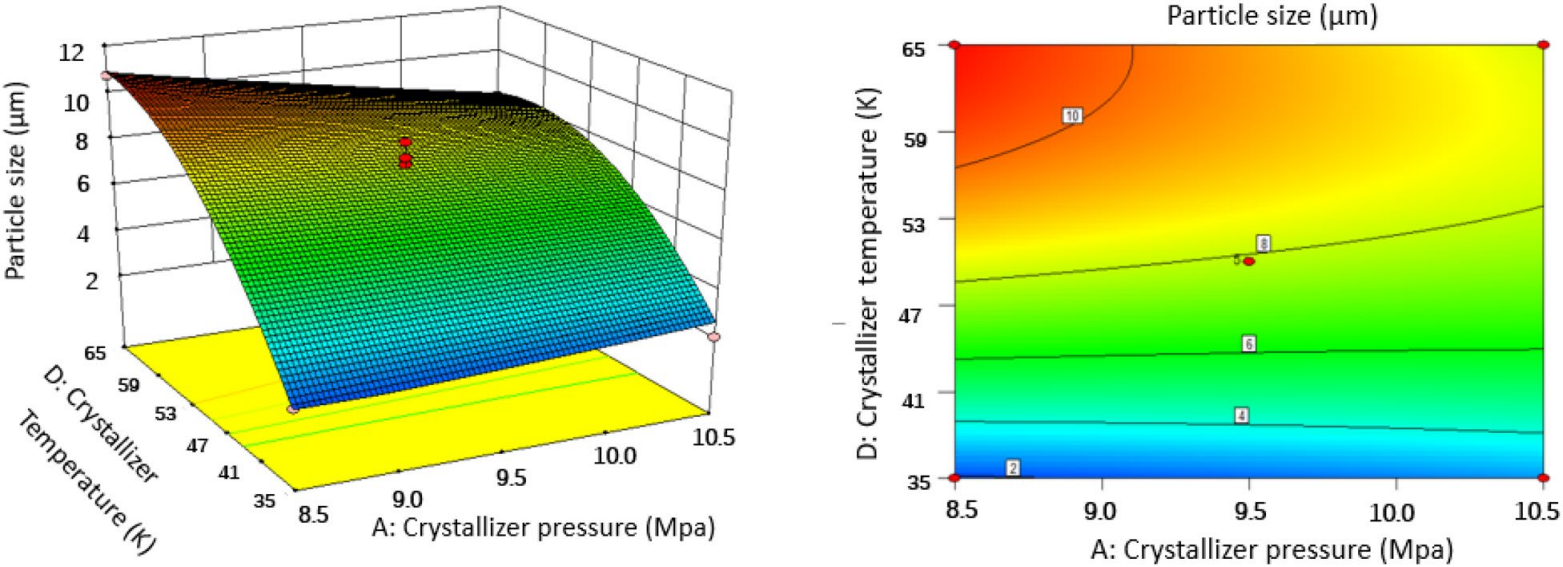
3D response surface and 2D contour plots showing the interaction between (**A**) crystallizer pressure and **(D**) crystallizer temperature.

**Figure 9:**
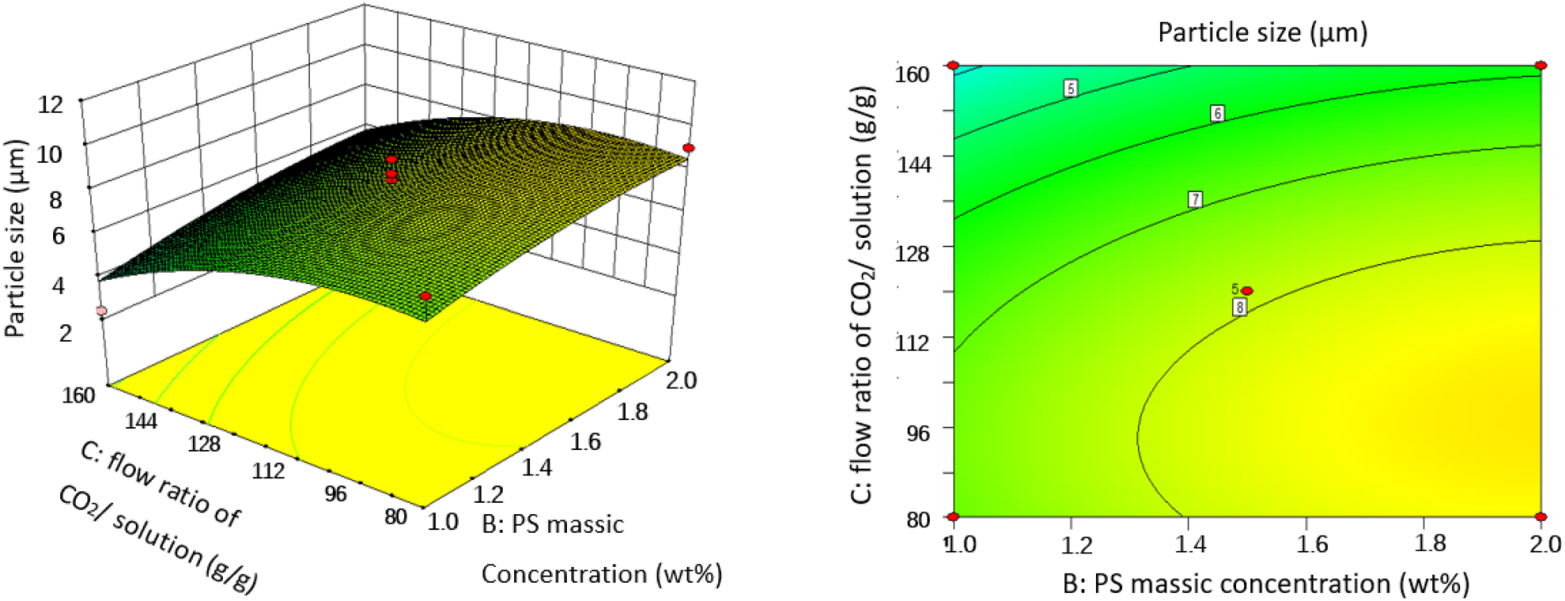
3D response surface and 2D contour plots showing the interaction between (**B**) PS massic concentration and (**C**) flow ratio of CO_2_/solution.

**Figure 10:**
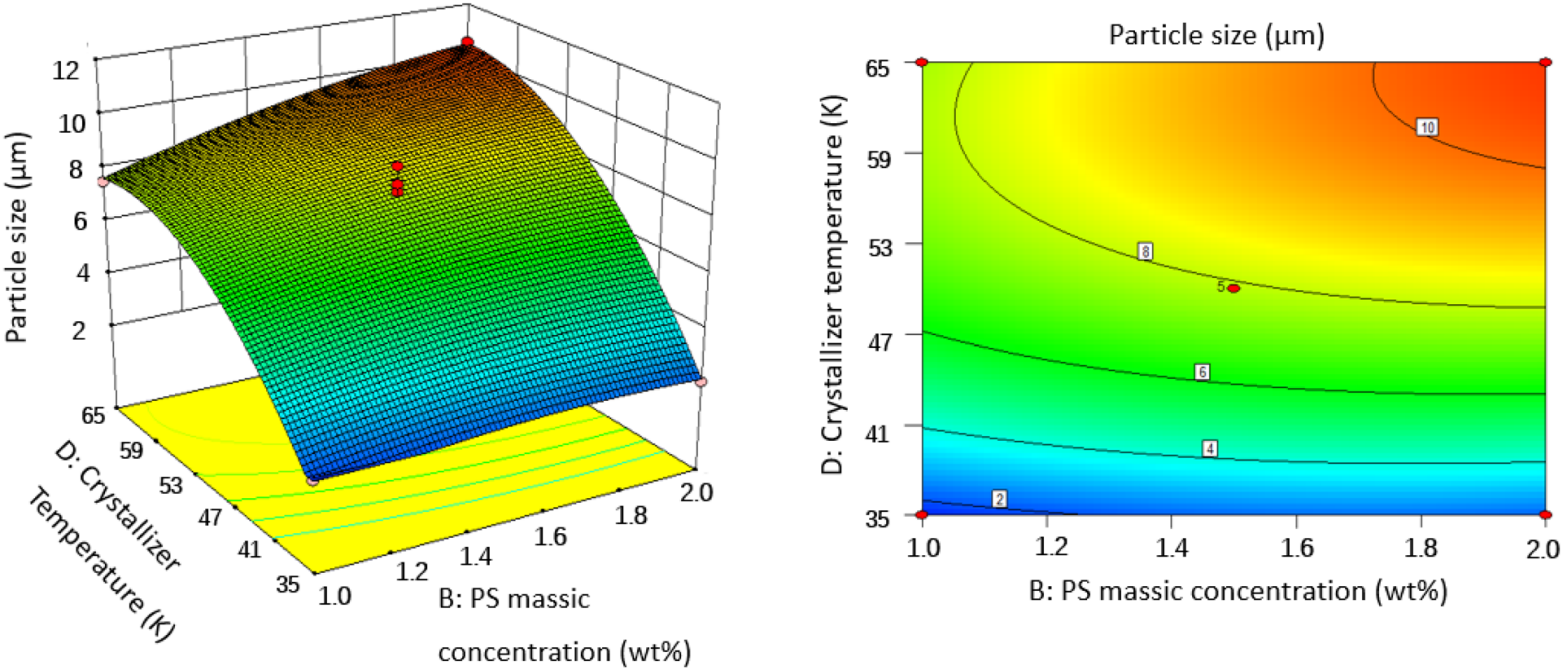
3D response surface and 2D contour plots showing the interaction between (**B**) PS massic concentration and (**D**) crystallizer temperature.

**Figure 11:**
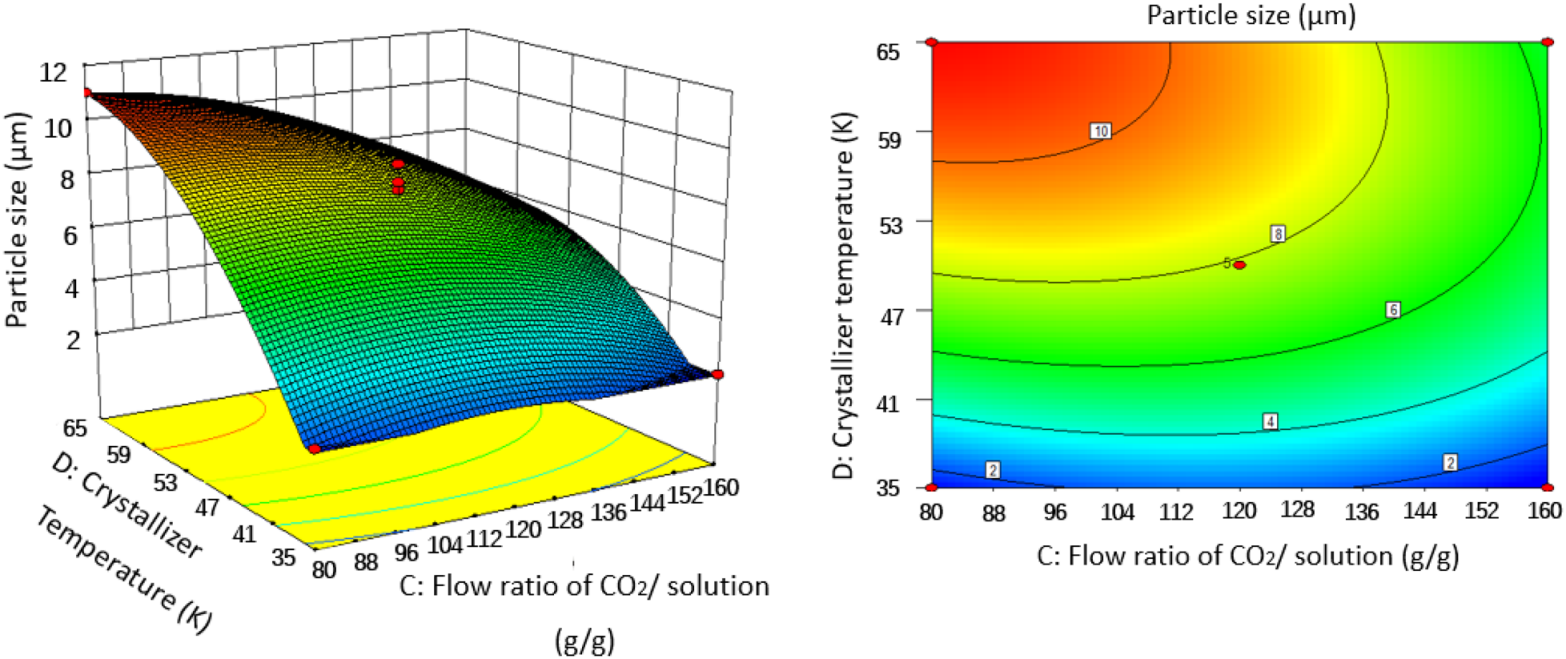
3D response surface and 2D contour plots showing the interaction between (**C**) flow ratio of CO_2_/solution and (**D**) crystallizer temperature.

In order to gain a better understanding of their interactions with PS particle size, three-dimensional (3D) response surface plots and two-dimensional (2D) contour plots for the measured responses were formed based on the regression equation [Eq. (2)]. Two variables were maintained at set level on account of the regression model with four independent variables in our study. The response surface analysis plots for particle size of microparticles are illustrated in Figs. 6, 7, 8, 9, 10 and 11.

#### Effect of crystallizer pressure

According to the optimization process in “Optimization of SAS process by BBD” section and Fig. 5, crystallizer pressure does not have a great impact on the particle size with the plateau like curve and the highest *P* value of 0.1934 compared with other parameters. Furthermore, crystallizer pressure also influenced the characteristics of PS particles. It was found that an increase in crystallizer pressure from 8.5 to 10.5 MPa induced a slight decrease on PS particle size by comparing runs 8 and 25, or runs 10 and 27, at a constant temperature of 323 K, combining with Figs. 6, 7 and 8. It can be explained in the aspect of nucleation and growth of particles. Density and viscosity of CO_2_ increase and diffusivity decreases considerably as the pressure increases. Thus, mass transfer between droplets and the surrounding is hindered and the average lifetime of droplets increase, leading to low supersaturation, low nucleation and large crystal particles. In addition, from the aspect of atomization, at high pressure, CO_2_ velocity is higher, so the jet break-up and turbulence after the solution mixed with CO_2_ are enhanced and the surface tension of a droplet is reduced. These phenomena lead to an evaporation rate of solvent higher than the rate of CO_2_ diffusion into the solvent and smaller crystal particles are obtained. Therefore, the effect of crystallizer pressure on the PS particle size may be case to case due to the opposite effect of mass transfer and atomization. This effect is evident in some papers published by Jeong et al.^21^ and Pérez de Diego et al.^29^. Since we observed a decrease in particle size with increasing pressure, so the atomization seems to be dominant ^30^.

#### Effect of PS massic concentration

The effect of PS massic concentration on PS particle size had been studied. *P* values given in Table 3 show that PS massic concentration greatly influences the PS particle size. As can be seen in Figs. 6, 9 and 10, PS massic concentration has a negative impact on particle size as it increasing from 1.00 to 2.00 wt% (runs 10 and 20, runs 18 and 26) during the SAS process. As explained previously, when PS massic concentration increases, the mass transfer rate and supersaturation decrease due to the increase of density and viscosity of CO_2_, and the decrease of atomization and jet breakage. The decrease of nucleation number and crystal growth become the main mechanism, leading to larger particles^31^. Meanwhile, when PS massic concentration increases, enough solute molecules around the nucleus facilitate them to grow. The same effect was reported by Reverchon et al.^32^, and they concluded that increasing the concentration of the solution resulted in larger particle size.

#### Effect of flow ratio of CO_2_/solution

Effects of flow ratio of CO_2_/solution varying at 92, 138 and 185 g/g corresponding respectively to CO_2_ mole fraction of 0.995, 0.996 and 0.997 on PS particle size were exhibited in Figs. 7, 9 and 11, and obviously showed that flow ratio of CO_2_/solution had a positive effect on particle size. As can be seen in Table 2 for runs 9 and 18, when flow ratio of CO_2_/solution increased from 92 to 185 g/g, the particle size decreased from 8.41 to 2.38 μm under the conditions of crystallizer pressure 9.5 MPa, PS massic concentration 1.00 wt% and crystallizer temperature 323 K. This may be because the relative content of solvent in the mixture of solution and SC-CO_2_ decreases when flow ratio of CO_2_/solution is large, which enhances the antisolvent effect of SC-CO_2_. Therefore, the formation of particles is mainly controlled by nucleation, and small particles are obtained. At the same time, the kinetic energy of atomization between CO_2_ and droplets also increases, resulting in high mass transfer, high supersaturation and small particles^33^.

#### Effect of crystallizer temperature

**Figure 12:**
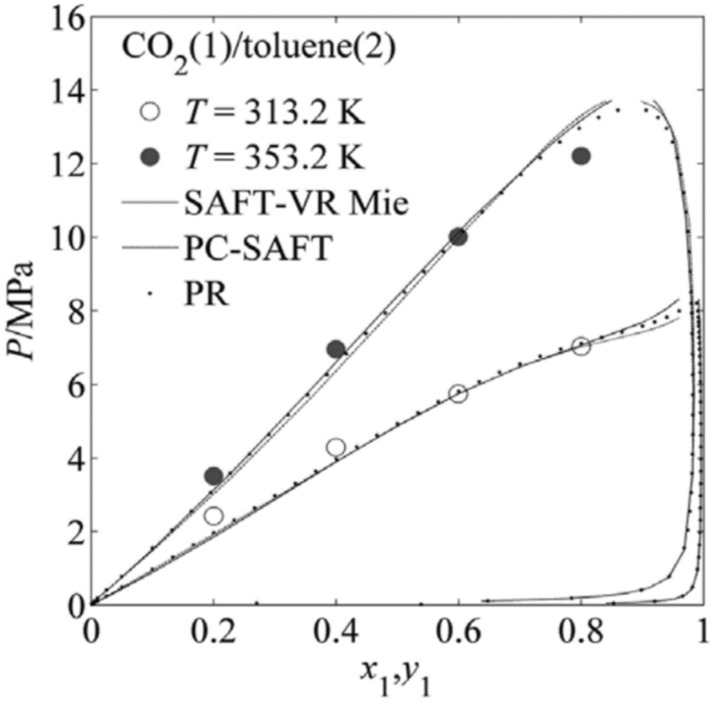
P–x–y phase diagram for CO_2_/toluene system at 313.2 and 353.2 K.

Variation of crystallizer temperature was used to investigate the effect on the particle size, while other parameters were kept at a certain level. It was observed from Figs. 5, 8, 10, and 11 that crystallizer temperature was the most significant parameter of response with the sharp curvature and the minimum *P* value of < 0.0001. As crystallizer temperature increased from 308 to 338 K, the particle size increased from 2.11 to 11.01 μm (see runs 5 and 6). The results showed that crystallizer temperature is the most important parameter affecting the particle size. The P–x–y phase diagram for CO_2_/toluene system at 313.2 and 353.2 K is displayed in Fig. 12^34^. It is evident that the experimental conditions in this paper are far away from the critical line with the decrease of temperature, leading to the increase of supersaturation of solid solute in the liquid solvent. Therefore, decreasing the temperature can improve the supersaturation of PS in the liquid solvent and smaller particles are obtained. Meanwhile, with the increase of crystallizer temperature, the kinetic energy of solute molecules increases, which accelerates the collision and aggregation of particles. According to Pérez de Diego et al.^29^, the lifetime of droplets depended on the diffusivity between CO_2_ and solvent. When the temperature increase, evaporation rate of the solvent is less than diffusion rate of CO_2_ in the solvent, leading to the increase of the droplet life and the initial droplet size^35^.

### Characterization of precipitated PS particles

#### Characteristics of PS particle morphology

**Figure 13:**
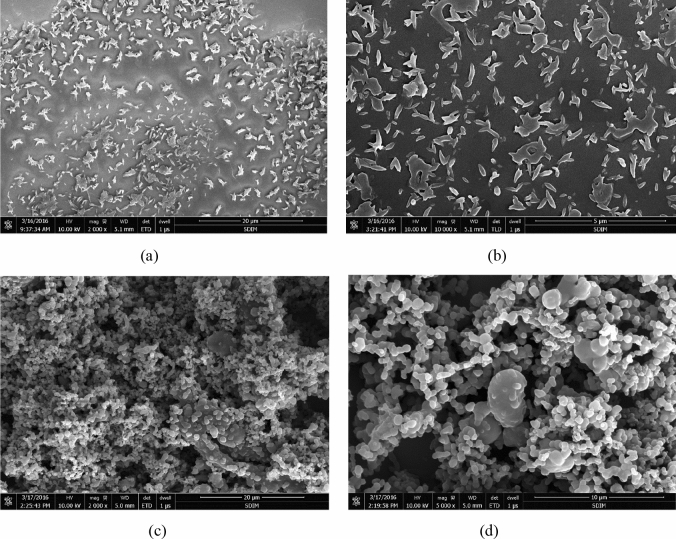
SEM micrographs of the precipitated particles at different conditions: (**a**) 9.5 MPa, 1.5%, 92 g/g, 308 K; (**b**) 9.5 MPa, 1%, 185 g/g, 323 K; (**c**) 9.5 MPa, 1.5%, 185 g/g, 308 K; (**d**) 8.5 MPa, 1.5%, 138 g/g, 308 K.

From the SEM images Fig. 13a, b, it was found that PS particles prepared by SAS process are pod-like with particle size between 1 and 2 μm for some operating conditions among the investigated range. A reason for this morphological characteristic may be that crystals grow in an unbalanced state with large surface free energy, which causes the crystals to grow along some edges or top angles, thus forming pod-like shaped particles. In contrast, many spherical agglomerated particles have uniform size, as shown in Fig. 13c, d, and their sizes are close to the optimum result. Since smaller particles possess large surface free energy and live in an unstable state, they are more likely to aggregate to reach to a stable state.

#### Characterization of particles size distribution

**Figure 14:**
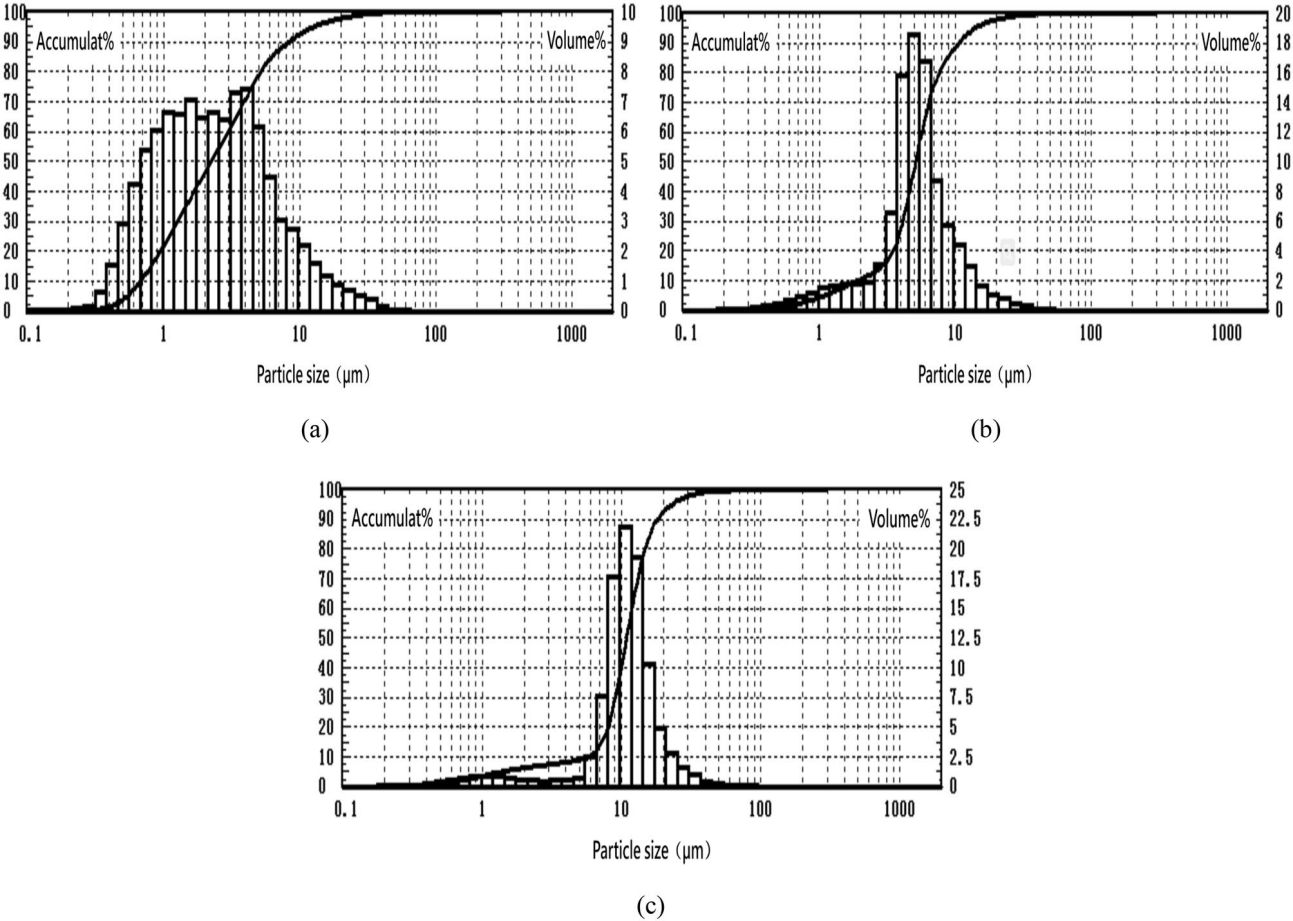
Particle size distribution of particles obtained at different conditions: (**a**) 9.5 MPa, 2%, 185 g/g, 323 K; (**b**) 9.5 MPa, 1.5%, 138 g/g, 323 K; (**c**) 9.5 MPa, 1.5%, 92 g/g, 338 K.

Particles size distributions measured by Laser Particle Size Analyzer at different conditions are shown in Fig. 14. According to the Table 2, the particle size under these three conditions are respectively 3.84, 6.05 and 11.01 μm, and the corresponding particle size distribution spans are 3.421, 1.699 and 1.146. Combining with Fig. 14, we can see that particles present a narrow size distribution with the increase of particle size. This may be due to the fact that, small particles are more likely to aggregate to a stable state, thus presenting an uneven size distribution. This is also consistent with the results of SEM images.

## Conclusions

In this study, PS particles ranging from 1.08 to 11.01 μm were successfully prepared by SAS process, and the effects of crystallizer pressure, PS massic concentration, flow ratio of CO_2_/solution and crystallizer temperature on the size and the distribution of the precipitated PS particles were systematically studied. Analysis of experimental results by the BBD-RSM demonstrate that the crystallizer temperature was the most significant factor on particle size, followed by flow ratio of CO_2_/solution and PS massic concentration, and crystallizer pressure was the slightest significant factor. The particle size increases with the increase of crystallizer temperature. The PS particles with a size of 2.78 μm and a narrow size distribution were prepared under the optimum conditions of crystallizer pressure 9.8 MPa, PS massic concentration 1.6 wt%, flow ratio of CO_2_/solution 140 g/g and crystallizer temperature 309 K. The current study showed that it was feasible to produce PM2.5 standard aerosols by SAS.

## Supplementary information

Supplementary information
