# Supplementary material for: Optimization of process parameters for preparation of polystyrene PM2.5 particles by supercritical antisolvent method using BBD-RSM
Source: Sci Rep. 2020 Jul 7;10:11187. doi: 10.1038/s41598-020-67994-4 (PMC7341738; doi:10.1038/s41598-020-67994-4)
Supplement: Supplementary file 1 — Supplementary information [file 41598_2020_67994_MOESM1_ESM.pdf]

Optimization of process parameters for preparation of polystyrene PM2.5 particles by supercritical antisolvent method using BBD-RSM

Zhuo Zhang[1], Qingling Li[1], Bo Guo[2], Shouzhong Zhang[2], Sen Zhang[3], Dedong Hu[1*]

[1] School of Electromechanical Engineering, Qingdao University of Science and Technology, Qingdao 266061, China
[2] Shandong Institute of Metrology, Jinan 250014, China
[3] Shandong Zhifu District Market Supervision Administration, Yantai 264001, China

Contents

1 Preliminary experiments 1

1.1 Analyse 2

1.2 Supplementary Reference 3

2 TGA analyse of toluene residual amount in BBD experiment 4

1. Preliminary experiments

Table 1 Preliminary experiments parameters

Run	Crystallization temperature （K）	Crystallizer pressure （MPa）	PS massic concentration （wt%）	Feed rate of CO_2 (l/min)	Injection rate of solution (ml/min)	Nozzle diameter (μm)	Solvent	Results	Reference
1	295	8	5	10	10	50	Toluene	Nozzle clogged	[1]
2	308	10	1.0	4	10	100	Toluene	Supplementary Figure 1	[2]
3	313.2	10	0.25	15	10	250	Toluene	hardly to collect	[3]
4	343.2	8.5	4	15	10	100	Toluene	Supplementary Figure 2	[3]

1.1 Analyse

The nozzle type is always considered as one of the critical parameters in SCF techniques. Therefore, different nozzle diameters were examined (50, 100, 250 μm) in our preliminary experiments. We found that small nozzle diameter will cause nozzle blockage, and large nozzle diameter will cause particle collection difficulty. We therefore decided to seleted the nozzle diameter with 100 μm.

Relevant references and literatures [2-4] on the application of SAS process demonstrated that lower concentrations are helpful for reducing the particle size and for narrowing the size distribution. Hence, we set the PS massic concentration at a relatively low value of 1.0, 1.5, 2.0 wt%. Based on the above literatures [1-3], we conclude that their experimental temperature range is between 295 and 343 K. In order to ensure the mixed fluid is in a supercritical state, we selected the temperature at 308, 323 and 338 K, respectively. In addition, the operating pressure is generally selected around 10 MPa in

supercritical range for the sake of satisfying the mass production levlel. Also, various experiments [5-8] have been conducted to study SAS process around 10 MPa in recent years. As the PS massic concentration is relatively low, we fixed the solution rate at 10 ml/min in order to collected the powders as soon as possible. It is seen from the CO_2/toluene phase diagram [9] that when the pressure is from 8.5 to 10.5 MPa and the temperature is from 308 to 338 K, only when the mole fraction of CO_2 is close to 1 can the CO_2/toluene binary system be in a supercritical state, and satisfys the prapering conditions of PS particles. Therefore, we control the flow ratio of CO_2 / solution to 92, 138 and 185 g/g and the corresponding CO_2 mole fraction was 0.995, 0.996 and 0.997, respectively.

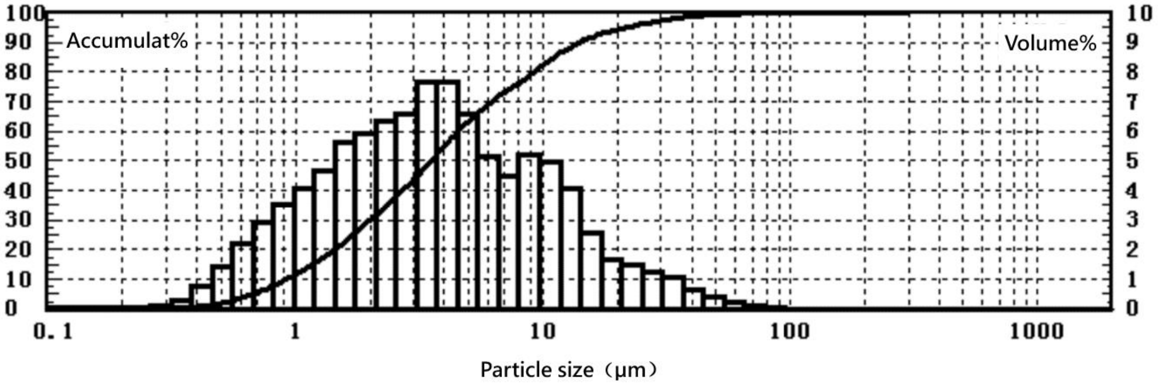

Supplementary Figure 1. Particle size distribution of particles obtained at preliminary experiment 6

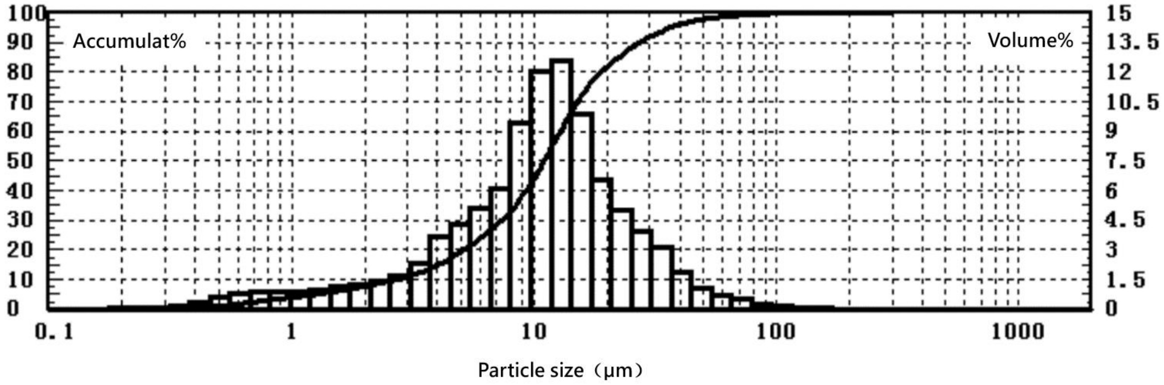

Supplementary Figure 2. Particle size distribution of particles obtained at preliminary experiment 9

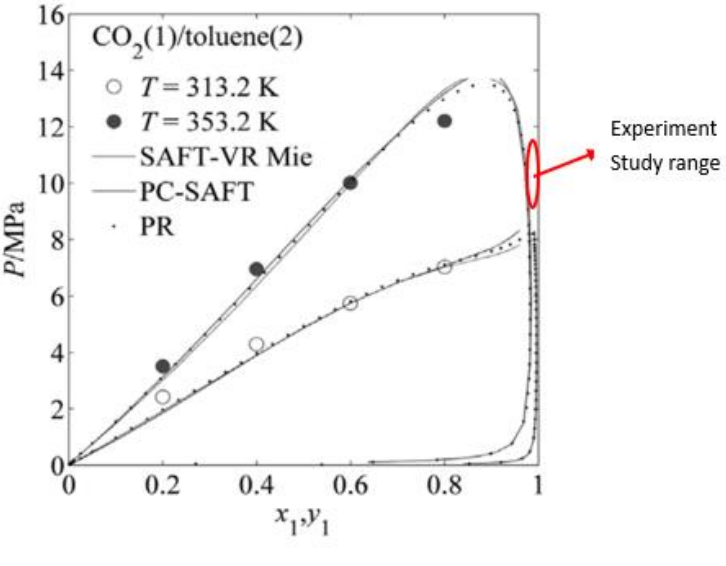

Supplementary Figure 3. P-x-y phase diagram for CO_2/toluene system at 313.2 and 353.2 K

1.2 Supplementary Reference

[1] Dixon D J, Johnston K P. Formation of Microporous Polymer Fibers and Oriented Fibrils by precipitation with a Compressed Fluid Antisolvent. J. Appl. Polym. Sci. 50(11): 1929-1942 (1993).

[2] Jeong H H, Yoo K P, Lim J S. Preparation of Polystyrene submicron particles using ASES process in supercritical carbon dioxide. J. Indus. & Eng. Che. 14(1): 77-83 (2008).

[3] Chang S C, Lee M J, Lin H M. Nanoparticles formation for metallocene catalyzed cyclic olefin copolymer via a continuous supercritical anti-solvent process. J. Supercrit. Fluid. 40(3): 420-432 (2007).

[4] Bakhbakhi Y, Asif M, Chafidz A. Supercritical antisolvent synthesis of fine griseofulvin particles. Adv. Powder Technol. 24(6): 1006-1012 (2013).

[5] Oliveira D. A, Mezzomo N, Gomes C, Ferreira S. R. S. Encapsulation of passion fruit seed oil by means of supercritical antisolvent process. J. Supercrit. Fluid. 129: 96–105 (2017).

[6] Costa M. S, Duarte A. R. C, Cardoso M. M., Catarina M. M. Duarte Supercritical antisolvent precipitation of PHBV microparticles. Int. j. pharmaceut. 328: 72–77 (2007).

[7] Marco I. D, Rossmann M, Prosapio V, Reverchon E, Braeuer A. Control of particle size, at micrometric and nanometric range, using supercritical antisolvent precipitation from solvent mixtures: Application to PVP. Chem. Eng. J. 273: 344-352 (2015).

[8] Petit-Gas T, Boutin O, Raspo I, Raspo, E. B. Role of hydrodynamics in supercritical antisolvent processes. J. Supercrit. Fluid. 51(2): 248-255 (2009).

[9] Nascimento F. P, Paredes M. L.L, Bernardes A. P. D, Pessoa F. L. P. Phase behavior of CO_2 /toluene, CO_2 /n-decane and CO2/toluene/n-decane: Experimental measurements and thermodynamic modeling with SAFT-VR Mie equation of state. J. Supercrit. Fluid. 154: 104634 (2019).

2. TGA analyse of toluene residual amount in BBD experiment

Thermogravimetric analyzer (TGA) was applied in this study to detect any residual toluene left in the particulate PS samples after the SAS process. During the TGA analysis, the samples were heated at a fixed heating rate (10 °C/min) from 37 to 400 °C in the atmosphere of N_2 and the flow rate of N_2 was 50 ml/min. The sample weight of experature 22, experature 24 and unprocessed PS were 5.5564, 5.7915, 8.7826 mg, respectively. The results of TGA analysis show that the unprocessed PS sample has a slight weight loss at about 100 °C and the weight loss reached to about 0.07 % at 150 °C, as depicted in Fig.4(c). This is due to the presence of water vapor in the air. The normal boiling point of toluene is 110 °C, which is far below the initial decomposition temperature of PS. The TGA analysis results as shown in Supplementary Figure 4 (a) and (b) also reveal that the PS samples after SAS processing has a slight weight loss by about 0.20 % and 0.23 % as temperature reaching 150 °C. Consequently, the residual toluene in the particulate product should be extremely low.

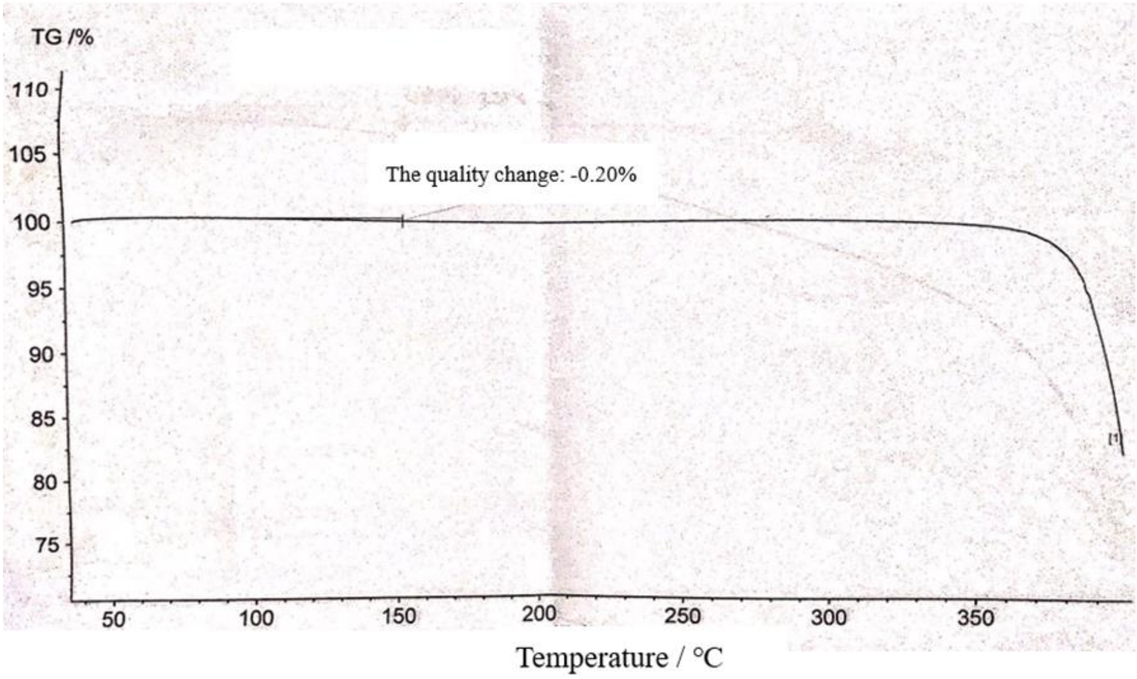

(a) Experature 22

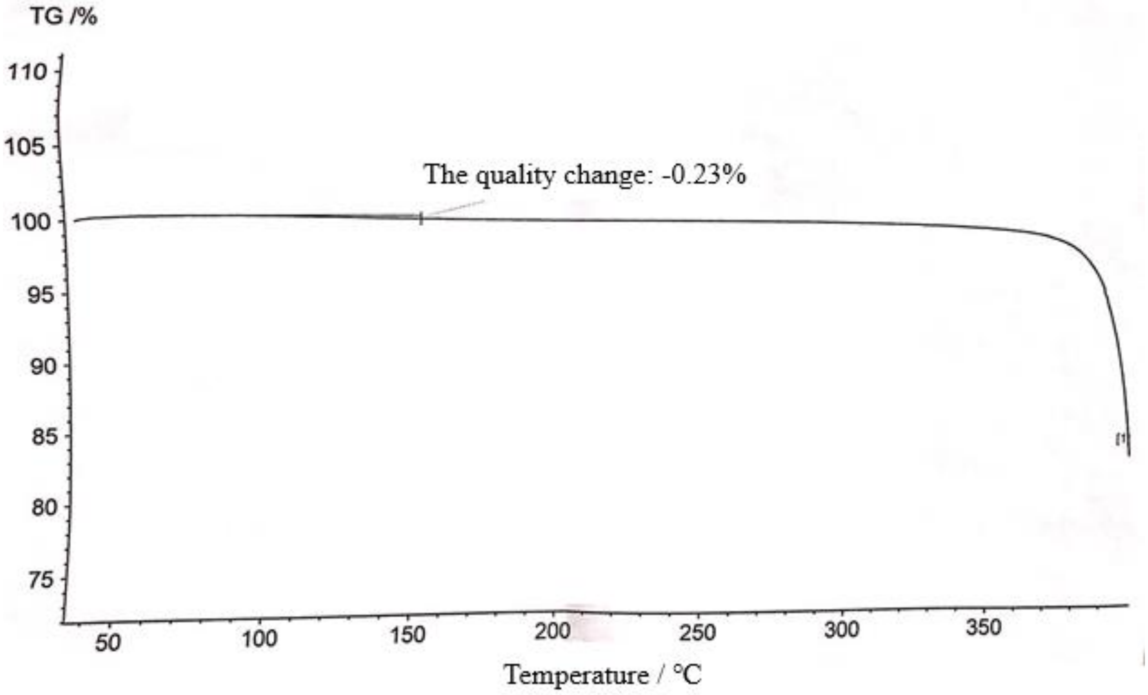

(b) Experature 24

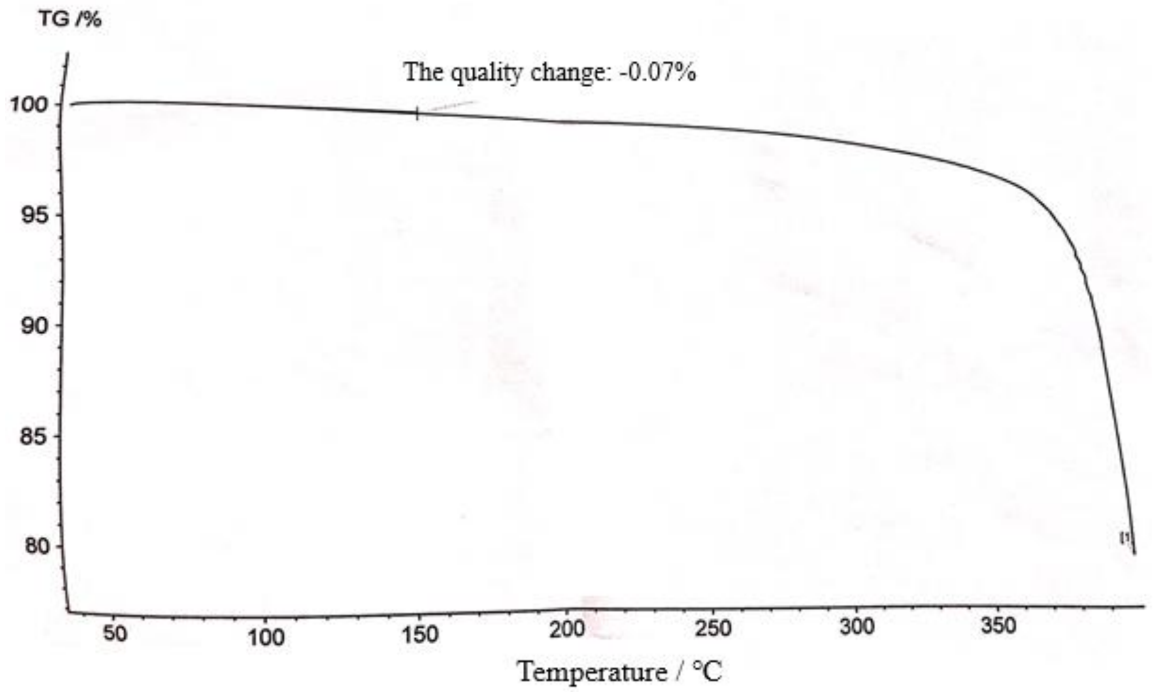

(c) Unprocessed PS

Supplementary Figure 4. TGA analysis results for the unprocessed PS and two samples after SAS processing
